# Genome-wide discovery of DNA polymorphisms among chickpea cultivars with contrasting seed size/weight and their functional relevance

**DOI:** 10.1038/s41598-018-35140-w

**Published:** 2018-11-14

**Authors:** Mohan Singh Rajkumar, Rohini Garg, Mukesh Jain

**Affiliations:** 1 School of Computational & Integrative Sciences, Jawaharlal Nehru University, New Delhi, 110067 India; 2 Department of Life Sciences, School of Natural Sciences, Shiv Nadar University, Gautam Buddha Nagar, Uttar Pradesh 201314 India; 3 National Institute of Plant Genome Research (NIPGR), Aruna Asaf Ali Marg, New Delhi, 110067 India

## Abstract

Seed size/weight is a major agronomic trait which determine crop productivity in legumes. To understand the genetic basis of seed size determination, we sought to identify DNA polymorphisms between two small (Himchana 1 and Pusa 362) and two large-seeded (JGK 3 and PG 0515) chickpea cultivars via whole genome resequencing. We identified a total of 75535 single nucleotide polymorphisms (SNPs), 6486 insertions and deletions (InDels), 1938 multi-nucleotide polymorphisms (MNPs) and 5025 complex variants between the two small and two large-seeded chickpea cultivars. Our analysis revealed 814, 244 and 72 seed-specific genes harboring DNA polymorphisms in promoter or non-synonymous and large-effect DNA polymorphisms, respectively. Gene ontology analysis revealed enrichment of cell growth and division related terms in these genes. Among them, at least 22 genes associated with quantitative trait loci, and those involved in cell growth and division and encoding transcription factors harbored promoter and/or large-effect/non-synonymous DNA polymorphisms. These also showed higher expression at late-embryogenesis and/or mid-maturation stages of seed development in the large-seeded cultivar, suggesting their role in seed size/weight determination in chickpea. Altogether, this study provided a valuable resource for large-scale genotyping applications and a few putative candidate genes that might play crucial role in governing seed size/weight in chickpea.

## Introduction

Chickpea seed is enriched in essential amino acids, which can serve as readily and cheaply available source of dietary proteins. Seed development is orchestrated via complex gene regulatory programs. The molecular mechanisms underlying seed development in model/crop plants have been analyzed via transcriptome analyses to some extent, and transcriptional regulatory networks and pathways associated with seed development have been revealed^1–6^. Seed size/weight is a major trait for crop productivity in cereals and legumes. Thus, it is extremely important to understand the molecular mechanisms underlying seed size/weight determination. Epigenetic mechanisms and hormonal signaling play important roles in seed size/weight determination^7–10^. The epigenetic signatures of the parents have been shown to be important for determining transcriptional activity in allele-specific-manner, which determines seed size/weight in Arabidopsis^7^. Auxin plays an important role in seed development from embryonic cell division to seed maturation^8,11^ and induces endosperm/seed growth^12^. Brassinosteroids have also been shown as positive regulator of seed size in Arabidopsis^9^. It has been demonstrated that transcription factors and kinases play important roles in seed size/weight determination^13–16^. Efficiency of photosynthetic assimilate accumulation have also been shown critical for determining seed size^17–19^.

Being one of the most important legume crop, it is important to recognize the molecular mechanisms underlying seed development and seed size/weight determination in chickpea. Recently, transcriptome analyses during seed development in small and large-seeded chickpea cultivars have suggested extended period of cell division and higher level of endoreduplication in the large-seeded cultivar as the possible factors that determine seed size/weight^6^. A few studies have also been carried out to identify the quantitative trait loci (QTLs) and genes involved in seed size/weight in chickpea via different approaches^20–25^. In soybean also, QTLs and underlying genes associated with seed weight/seed quality have been analyzed^26–31^.

Identification of genetic variations governing seed size/weight offers excellent opportunity to select candidate genes to manipulate seed size/weight. We identified DNA polymorphisms between two small and two large-seeded chickpea cultivars via whole genome resequencing. We integrated transcriptome data from our previous study^6^ to correlate DNA polymorphisms and differential gene expression^6^. To understand the functional relevance of DNA polymorphisms in seed size/weight determination, we analyzed the effect of DNA polymorphisms on differential expression of seed-specific genes, particularly, genes involved in cell growth and division based on gene ontology (GO) terms, genes encoding transcription factors and those associated with QTLs. Finally, a few putative genes exhibiting DNA polymorphisms in promoter, and non-synonymous/large-effect changes that showed higher expression at late-embryogenesis and/or mid-maturation stage(s) of seed development in the large-seeded cultivar, were identified. This study provides a valuable resource of genetic variations and a few putative candidate genes, which determine seed size/weight in chickpea.

## Results and Discussion

### Genome-wide identification of DNA polymorphisms

We selected two small-seeded (Himchana 1 and Pusa 362 with 100 seed weight of 13.15 ± 0.15 g and 23.47 ± 1.14 g, respectively) of desi-type and two large-seeded (JGK 3 and PG 0515 with 100 seed weight of 53.3 ± 1.48 g and ~58.96 ± 2.40 g, respectively) of kabuli-type chickpea cultivars for genome resequencing to identify DNA polymorphisms. A total of 189–213 million 90-nt long high quality paired-end reads for each cultivar were generated via sequencing of genomic DNA using Illumina platform. About 116–131 million reads for each cultivar mapped uniquely to the chickpea genome (Supplementary Table S1) that covered ~84% of the reference genome (Supplementary Table S2). Only the nucleotides with read depth of ≥10 were considered for downstream analysis (Supplementary Fig. S1). A polymorphism call rate of ≥90% was used to identify DNA polymorphisms. Using these criteria, we identified a total of 355155 SNPs (single nucleotide polymorphisms), 10340 MNPs (multi-nucleotide polymorphisms), 32355 InDels (insertions and deletions) and 24847 complex variants among the small and large-seeded chickpea cultivars in one to one comparison (Supplementary file 1). In this study, we analyzed non-canonical DNA polymorphisms, such as multi-nucleotide polymorphisms (MNPs) and complex variants, where MNPs represent occurrence of at least two SNPs consecutively and complex variants represent occurrence of either SNPs or MNPs with InDels consecutively. Among the four types of DNA polymorphisms, SNPs were most frequent than other types of DNA polymorphisms (Supplementary Fig. S2). The number and frequency of InDels were higher than complex variants and MNPs. In different chromosomes, varying frequencies of DNA polymorphisms were observed. Chromosome 4 harbored highest number and frequency of DNA polymorphisms, while least frequency of DNA polymorphisms was observed in chromosome 5 (Supplementary Fig. S2).

**Figure 1:**
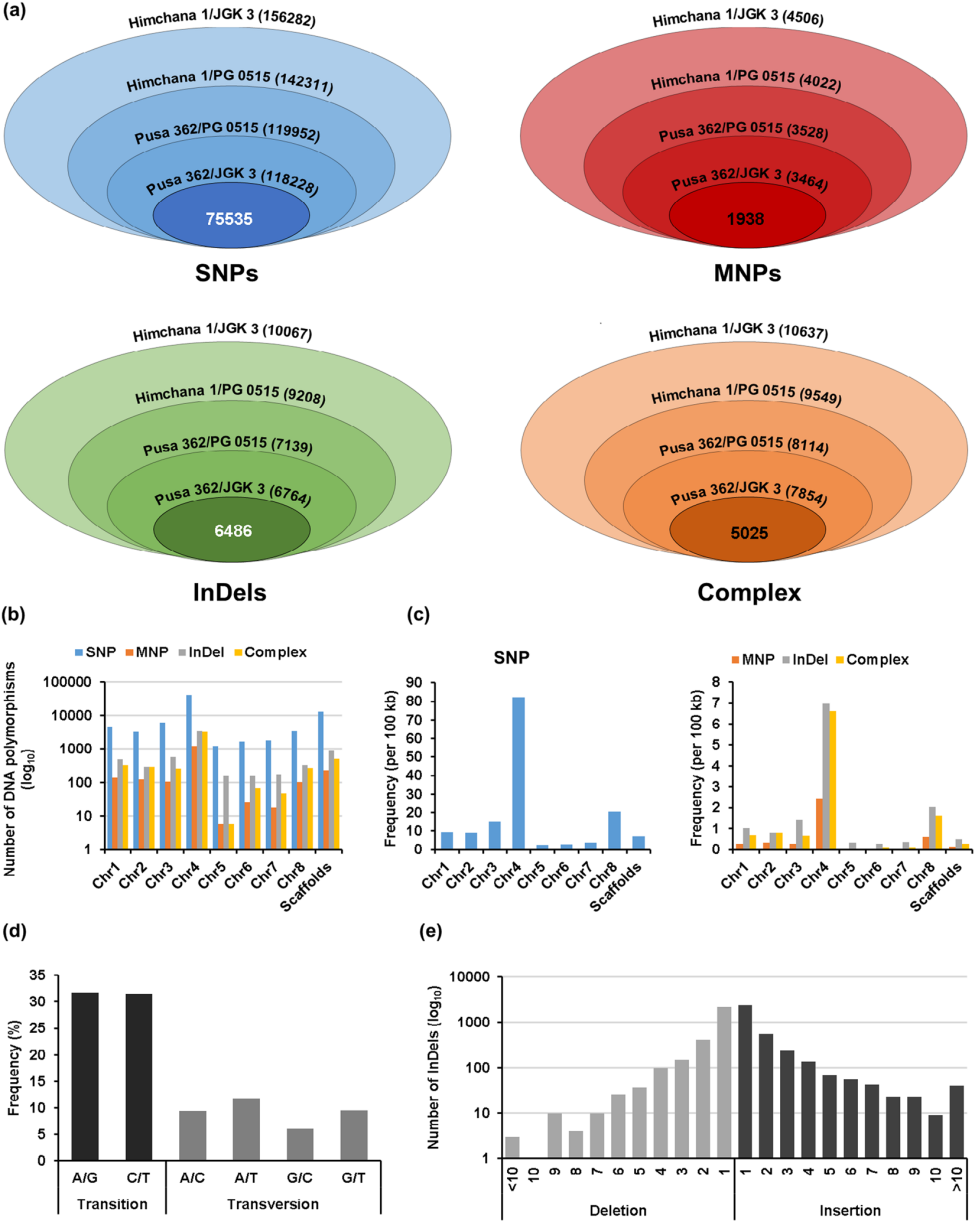
DNA polymorphisms differentiating small and large-seeded chickpea cultivars. (**a**) Number of DNA polymorphisms between small and large-seeded chickpea cultivars are shown in concentric rings as mentioned in the respective rings. Innermost circles represent the number of DNA polymorphisms identified between the two small and two large-seeded chickpea cultivars (Himchana 1-Pusa 362/JGK 3-PG 0515). (**b**,**c**) Number (**b**) and frequency (**c**) of DNA polymorphisms identified between the two small and two large-seeded chickpea cultivars on different chickpea chromosomes and scaffolds are shown in bar graphs. (**d**) Bar graph showing the frequency of transition and transversion substitutions. (**e**) Length distribution of insertions and deletions is shown in the bar graph.

To identify genetic variants that may determine seed size/weight, DNA polymorphisms that differentiate both the small and large-seeded chickpea cultivars, were identified. We identified a total of 75535 SNPs, 1938 MNPs, 6486 InDels and 5025 complex variants that differentiated both small and large-seeded chickpea cultivars (Fig. 1a). The lists of these DNA polymorphisms are provided in Supplementary file 2. Number and frequency of SNPs was highest (14.19 per 100 kb) followed by InDels (1.22 per 100 kb), complex variants (0.94 per 100 kb) and MNPs (0.36 per 100 kb) (Fig. 1b,c). The higher frequency of SNPs in comparison to InDels have been observed in previous studies too^23,32,33^. We found that both number and frequency of DNA polymorphisms was highest in chromosome 4 (Fig. 1b,c). In contrast, chromosome 5 harbored least number of DNA polymorphisms. We detected only 2.54 SNPs per 100 kb in chromosome 5 as compared to 82.19 SNPs per 100 kb in chromosome 4. Likewise, highest frequency of InDels, MNPs and complex variants was observed in chromosome 4 and lowest frequency in chromosome 5 (Fig. 1b,c).

### Analysis and annotation of DNA polymorphisms

The DNA polymorphisms identified between both the small and large-seeded chickpea cultivars were annotated based on their position and impact on protein function/integrity. About 63.3% of total SNPs represented transitions and the remaining 36.7% represented transversions. There was no bias between A/G and C/T base pair substitutions within transitions (Fig. 1d). However, frequency of A/T transversion was highest (11.77%) and G/C transversion was least (6.09%). It is generally observed that frequency of transitions is more than the transversions^32–35^. The higher frequency of transition is due to frequent occurrence of tautomeric shifts and deamination^36,37^ and they oftenly create synonymous substitutions^38^.

The size of insertions ranged from 1–20 nucleotides and deletions were in the range of 1–10 nucleotides in length. However, most (69.94%) of the insertions and deletions were of single nucleotide only. Di and tri-nucleotide insertions and deletions accounted for 14.97% and 6% of the total InDels, respectively (Fig. 1e). Higher frequency of smaller insertions and deletions have been observed in other studies too^34,35^. The total number of insertions (3570) was slightly higher than deletions (2916). Among larger InDels of ≥4-nt, total number of insertions (399) was significantly higher than deletions (191) (Fig. 1e).

**Figure 2:**
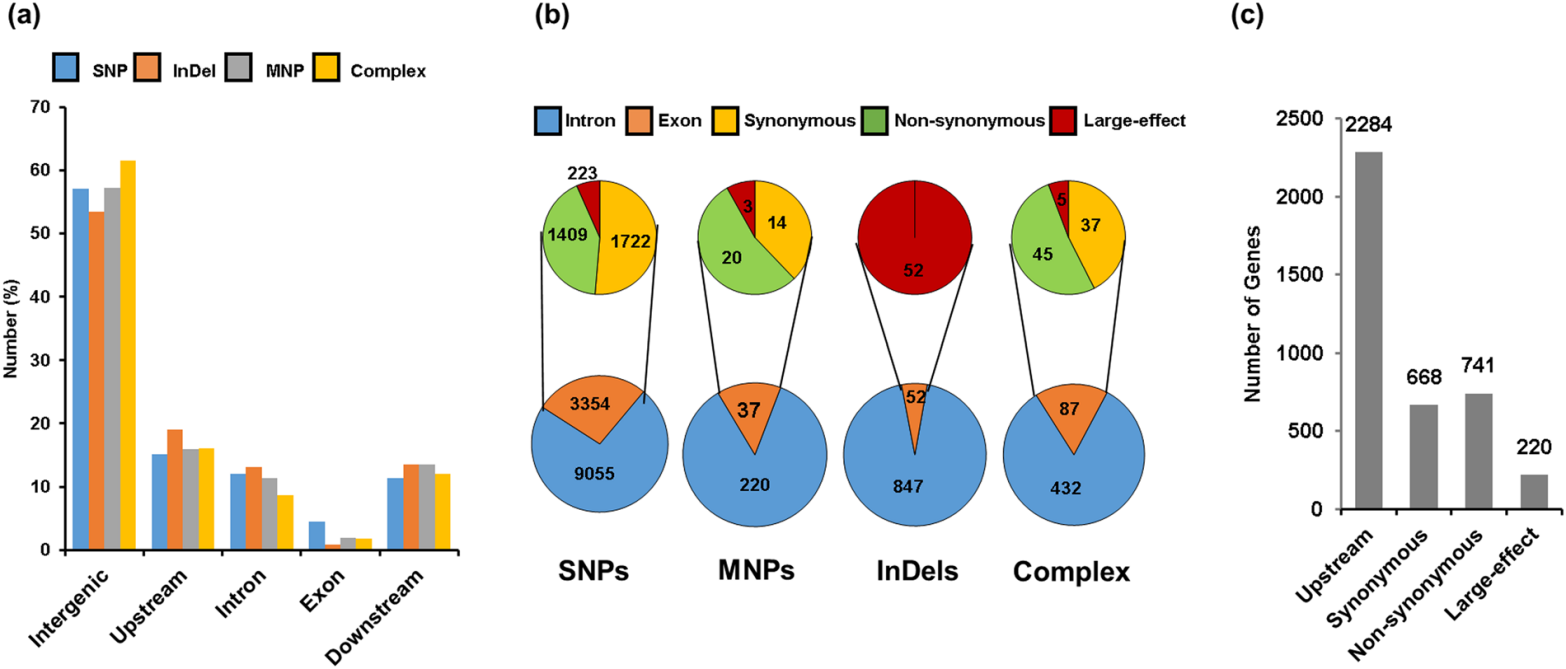
Annotation of DNA polymorphisms. (**a**) Percentage distribution of different types of DNA polymorphisms (SNPs, MNPs, InDels and complex) identified between the two small and two large-seeded chickpea cultivars in different genomic regions are shown in bar graphs. (**b**) DNA polymorphisms identified in the coding sequence (CDS) are classified into synonymous, non-synonymous and large-effect for different types of DNA polymorphisms (SNPs, MNPs, InDels and complex). (**c**) Number of genes harboring promoter (upstream), synonymous, non-synonymous and large-effect DNA polymorphisms are shown for all types of DNA polymorphisms.

The effect of DNA polymorphisms on gene expression is solely dependent on their genomic location. Therefore, we interrogated the presence of DNA polymorphisms in different genomic regions. We found that majority of the DNA polymorphisms (57.06% SNPs, 53.5% MNPs, 57.28% InDels and 61.53% complex variants) were located in the intergenic regions (Fig. 2a), which is in agreement with previous studies^32–35^. The number and frequency of DNA polymorphisms in promoter (2 kb upstream) was highest, intermediate in introns and least in exons (Fig. 2a). The higher frequency of DNA polymorphisms in intergenic regions may be due to high proportion of neutral mutations there. Therefore, we analyzed only those DNA polymorphisms, which were identified in the promoter regions and exons that may influence gene expression and protein functions, respectively.

Further, the DNA polymorphisms located in the exons were classified into synonymous, non-synonymous and large-effect (Fig. 2b). Large-effect refers to frame shift mutation, loss of start site, premature termination due to gain of stop codon and mutation at the splice junctions. The number of large effect DNA polymorphisms showing different effects on gene function has been given in Supplementary Table S3. InDels in exons were mostly associated with large-effect changes, while only 6.65%, 8.11% and 5.75% SNPs, MNPs and complex variants, respectively, exhibited large-effects (Fig. 2b). The overall higher frequency of SNPs over InDels may be due to selection against adverse effects of mutations caused by InDels on protein function.

DNA polymorphisms in the promoter region can have profound effect on gene expression via interfering with affinity/binding of transcription factors^39–41^. A total of 2284 genes harboring DNA polymorphisms in the promoter regions were detected (Fig. 2c). In addition, a total of 668, 741 and 220 genes harboring synonymous, non-synonymous and/or large-effect DNA polymorphisms, respectively, were detected (Fig. 2c). Previous studies have shown that DNA polymorphisms in the coding regions can have profound effect on gene expression/function, since they can disrupt the integrity of the encoded proteins^42–45^. Although synonymous DNA polymorphisms do not change the sequence of amino acids in the encoded proteins, alternative codon usage has been reported to modulate transcription^46,47^. Non-synonymous and/or large-effect DNA polymorphisms change the sequence of amino acids of the encoded protein and may exert large-effect due to incorporation of pre-mature stop codon, loss/gain of start site/splice junctions and frame-shift^42–45,48,49^. Further, we identified the genes harboring significantly high frequency of DNA polymorphisms in the genic regions, which may have implications on gene/genome evolution. Frequency of DNA polymorphisms per kb of genic regions was analyzed and represented via boxplot (Supplementary Fig. S3a). Frequency of DNA polymorphisms above the third quartile was considered to be significantly high (outliers). A total of 336 genes harbored high frequency (outliers) of DNA polymorphisms in the exonic regions (Supplementary Fig. S3b). Lesser number of genes (135) harbored significantly high frequency of DNA polymorphisms in the intronic regions. The DNA polymorphisms in introns are expected to exert mild effect on gene expression, unless they alter cryptic regulatory elements. Genes harboring significantly high frequency of DNA polymorphisms in exons constituted majorly synonymous (148 genes) followed by non-synonymous (125 genes) and large-effect (63 genes) DNA polymorphisms (Supplementary Fig. S3b). Functional categorization of these genes revealed enrichment of signal transduction (10%), post-transcriptional modification (8.43%), transcription (6.84%) and translation (5.94%) terms (Supplementary Fig. S4).

To validate SNPs identified in this study, we randomly selected 48 SNPs located in the 20 genes. Majority of them were lying in the promoter regions (60.42%) and exons (39.58%). We employed mass spectrometry to identify nucleotide bases at the selected polymorphic sites in the four chickpea cultivars (Himchana 1, Pusa 362, JGK 3 and PG 0515). Nucleotide bases identified via mass spectrometry in the polymorphic sites were compared with the nucleotide bases revealed by genome resequencing. Out of the 192 nucleotide bases that corresponded to 48 SNPs in the four cultivars, at least 179 nucleotide bases were same in both genome resequencing and mass spectrometry analyses. Overall, ~95% of the nucleotide bases detected via genome resequencing were validated using mass spectrometry (Supplementary Table S4).

### Identification and analysis of seed specific genes harbouring DNA polymorphisms

**Figure 3:**
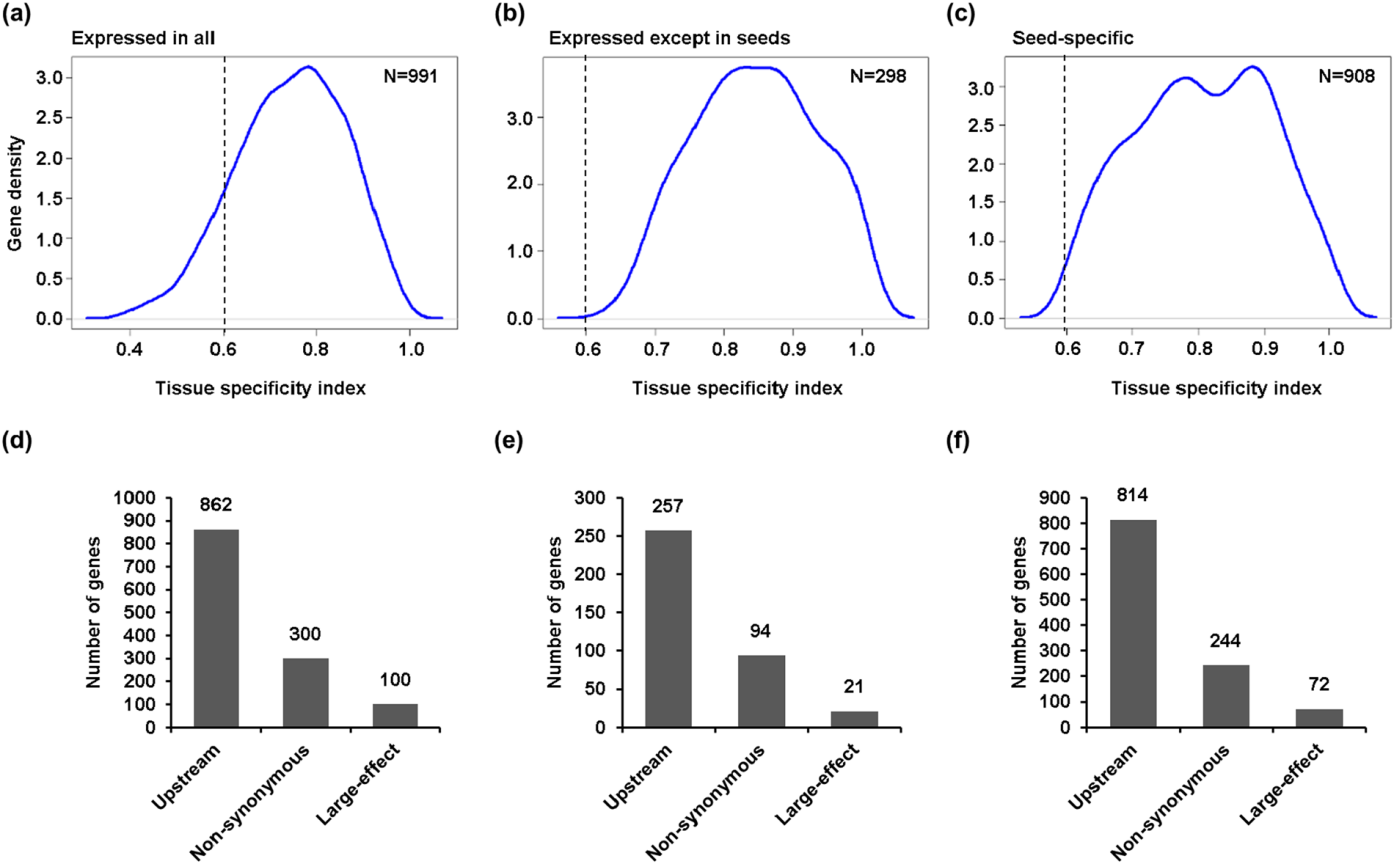
Identification of seed-specific genes harboring DNA polymorphisms. Tissue specificity index for the genes harboring DNA polymorphisms in their promoter and/or genes that harbor large-effect/non-synonymous DNA polymorphisms, was determined in 29 different tissues, including seven stages of seed development each from two chickpea cultivars and 15 vegetative/floral tissues/organs. (**a**–**c**) Tissue specificity index of genes expressed throughout the plant tissues/organs/development stages (**a**), genes expressed except in seeds (**b**) and genes specifically expressed in seeds (**c**) are shown via kernel density plots. More than 0.6 tissue-specific index was used for identification of seed-specific genes. N indicates number of the genes. (**d**–**f**) Number of genes harboring DNA polymorphisms in their promoter and/or genes that harbor non-synonymous/large-effect DNA polymorphisms on the genes expressed throughout the plant (**d**), expressed except in seeds (**e**) and seed-specific genes (**f**), respectively, is shown in bar graphs.

Seed development and seed size/weight are orchestrated via complex gene regulatory networks^1–6^. We categorized genes harboring DNA polymorphisms in their promoter and/or genes that harbor large-effect/non-synonymous DNA polymorphisms into three sets based on their expression patterns using transcriptome data from 14 samples representing stages of seed development^6^ and 15 vegetative and/or floral tissues from the chickpea cultivar(s)^50^. First set included genes showing expression in all the tissues analyzed (Fig. 3a), second set comprised of those genes not expressed during seed development (Fig. 3b) and third set included genes showing specific expression during seed development stages (Fig. 3c). To analyse the plausible influence of DNA polymorphisms on seed size/weight determination, we considered genes specifically expressed during seed development in the subsequent analyses. Seed-specific genes were screened via calculating tissue specificity index among the 29 different stages/tissues. Genes showing higher expression at any seed stage and at least >0.6 tissue specificity index were considered as seed-specific genes (Fig. 3c). With these criteria, a total of 908 seed-specific genes harboring DNA polymorphisms in the promoter and/or large-effect/non-synonymous DNA polymorphisms were identified (Fig. 3c). Of these, 814 genes exhibited DNA polymorphisms in promoter, and 244 and 72 genes harboured non-synonymous and large-effect DNA polymorphisms, respectively (Fig. 3f).

**Figure 4:**
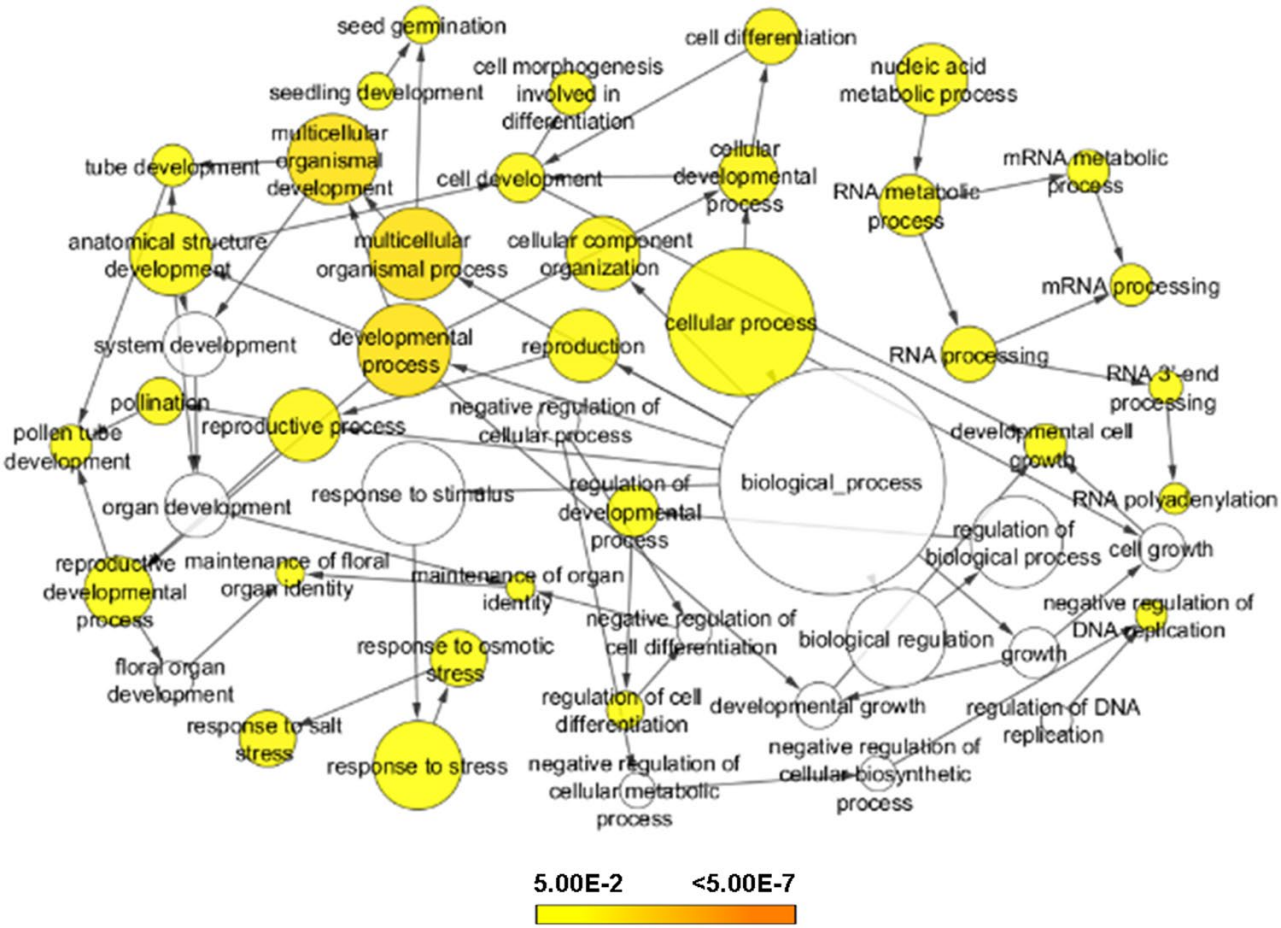
Gene ontology enrichment analysis of seed-specific genes harboring DNA polymorphisms in the promoter region and/or large-effect/non-synonymous DNA polymorphisms. Gene ontology (biological process) enrichment analysis of seed-specific genes harboring DNA polymorphisms in promoter and/or genes that harbor large-effect/non-synonymous DNA polymorphisms is shown. Scale represents *p*-value of enriched gene ontology (GO) terms.

Gene ontology (GO) enrichment analysis of the genes harboring DNA polymorphisms in promoter and/or genes that harbor large-effect/non-synonymous DNA polymorphisms was carried out. Genes harboring the DNA polymorphisms showed enrichment of cell development/differentiation/morphogenesis, RNA processing, response to stress and seed development related functional terms (Fig. 4). Presence of development and cell growth related genes among the polymorphic genes indicated that DNA polymorphisms may be an important factor responsible for determining seed size/weight in chickpea.

### Influence of DNA polymorphisms on differential gene expression determining seed size/weight in chickpea

**Figure 5:**
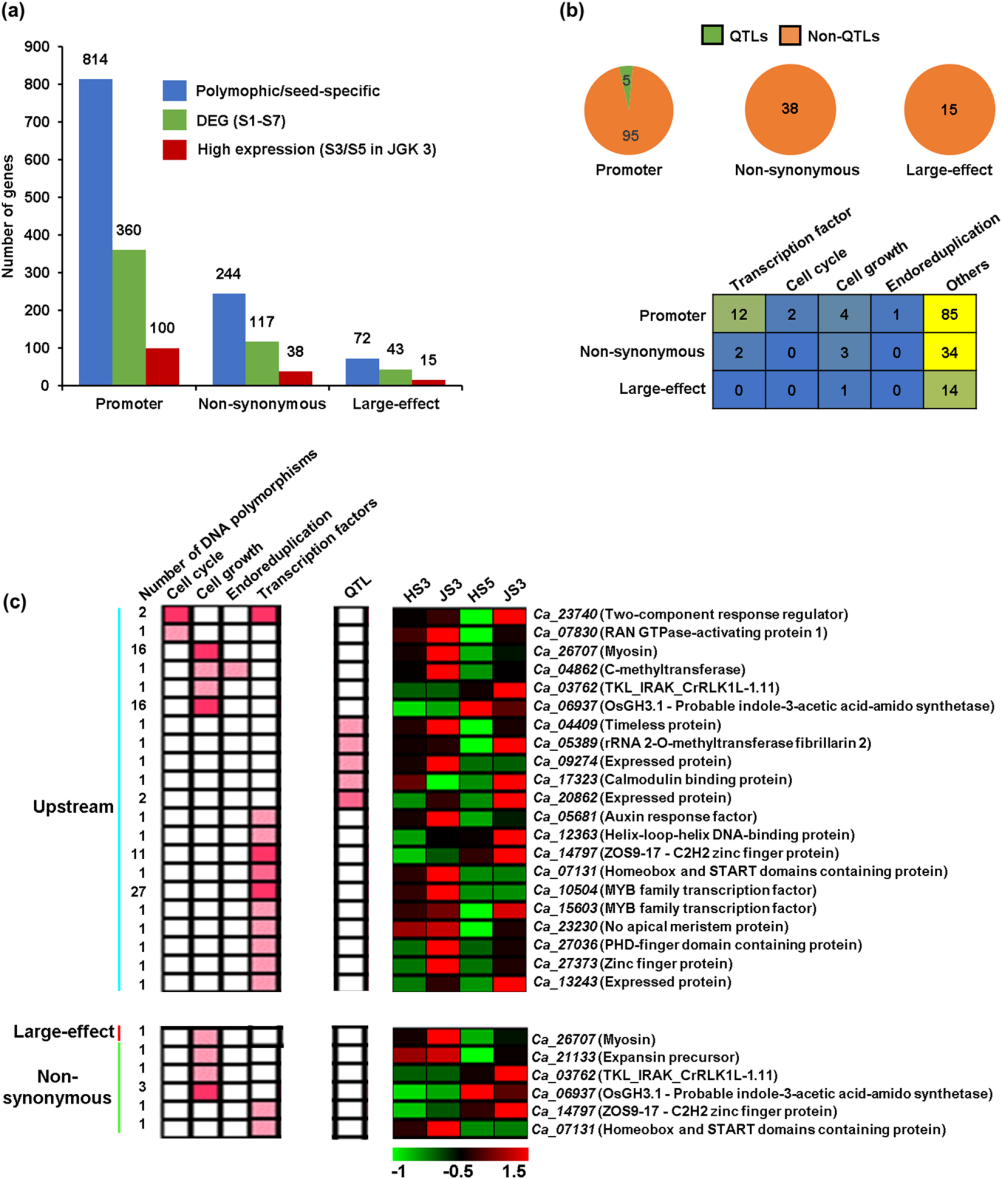
DNA polymorphisms associated with differential gene expression and seed size determination. (**a**) Number of seed-specific genes harboring promoter, non-synonymous and large-effect DNA polymorphisms that exhibit differential expression between the small and large-seeded cultivars at any of the seven stages of seed development, and higher expression at S3 and/or S5 stages of seed development in the large-seeded cultivar is shown. (**b**) Number of QTL-associated and non QTL-associated genes that show high expression at S3 and/or S5 stages of seed development in JGK 3 and harbor DNA polymorphisms, are given in the pie chart. Genes showing higher expression at S3 and/or S5 stages of seed development in JGK 3 harboring promoter, non-synonymous and/or large-effect DNA polymorphisms are categorized into genes encoding transcription factor or those involved in cell cycle, cell growth, endoreduplication or other processes. (**c**) Heatmaps showing presence of DNA polymorphism(s) (promoter, non-synonymous and large-effect) and gene expression of QTL-associated genes, and those involved in cell cycle, cell growth, endoreduplication and genes encoding transcription factors. Scale represents *z*-score based on FPKM expression values. JS3, JS5, HS3 and HS5 represent S3 and S5 stages of seed development in JGK 3 (JS3 and JS5) and Himchana 1, (HS3 and HS5), respectively. Labels on the left side indicate number of DNA polymorphisms. Gene identifiers and gene names are given on the right side.

To examine the influence of DNA polymorphisms on gene expression associated with seed size/weight determination, we identified differentially expressed genes between small (Himchana 1) and large-seeded (JGK 3) chickpea cultivars during stages of seed development^6^ that harbor DNA polymorphisms in promoter and/or exons resulting into non-synonymous substitutions or large-effect changes. Our previous transcriptome analyses showed that differential gene expression at late-embryogenesis (S3) and/or mid-maturation (S5) stages of seed development determine seed size/weight in chickpea to a large extent^6^. Therefore, we focused on the genes that showed differential expression at S3 and/or S5 stages of seed development in the chickpea cultivars. A total of 100, 38 and 15 genes harboring promoter, non-synonymous and large-effect DNA polymorphisms, respectively, that showed higher expression at S3 and/or S5 stages of seed development in the large-seeded cultivar, were identified (Fig. 5a).

In chickpea, quite a few QTLs involved in seed size/weight have been identified using various approaches^20–25^. Based on the genomic co-ordinates available in the previous studies, we identified the genes located within these QTLs and examined the impact of DNA polymorphisms on their differential expression. At least five QTL-associated genes harboring DNA polymorphism(s) in the promoter region showed higher expression at S3 and/or S5 stages of seed development in the large-seeded cultivar (Fig. 5b). Further, we analyzed sets of genes involved in cell cycle, cell growth, endoreduplication and genes encoding for transcription factors (Fig. 5b). At least two, four, one and 12 genes belonging to cell cycle, cell growth, endoreduplication and transcription factors, respectively, harbored DNA polymorphisms in the promoter region. Four and two genes belonging to cell growth and transcription factors harbored large-effect/non-synonymous DNA polymorphisms, respectively (Fig. 5b,c).

Altogether, at least 22 genes showing higher expression in the large-seeded cultivar during S3 and/or S5 stages of seed development harbored DNA polymorphisms in their promoter regions and/or non-synonymous substitutions or large-effect changes (Fig. 5c). Among them, five genes were associated with QTLs, including a gene involved in DNA replication (*Ca_04409*, timeless protein). The importance of timeless proteins in regulating replication folk rotation is well understood^51,52^. Among the genes involved in cell growth and division, *Ca_23740* (two-component response regulator, putative), *Ca_07830* (RAN GTPase-activating protein 1, putative), *Ca_04862* (C-methyltransferase), *Ca_26707* (myosin, putative), *Ca_03762* (TKL_IRAK_CrRLK1L), *Ca_06937* (OsGH3.1 - probable indole-3-acetic acid-amido synthetase) and *Ca_21133* (expansin precursor), were included. Two-component mediated regulation of cell division in meristems^53,54^, requirement of RanGTPs for mitotic cell division in female gametophyte^55,56^, role of sterol methytransferase mediated induction of ectopic endoreduplication^57,58^ and the role of myosin in phragmoplast assembly during cell division^59,60^ have been reported in different studies. Previous studies have shown that receptor like kinases^61,62^ and auxin signaling play crucial roles in cell division and cell expansion in the meristematic tissue^63,64^. Further, 11 genes encoding for transcription factors, such as *Ca_05681* (auxin response factor), *Ca_12363* (bHLS, helix-loop-helix DNA-binding protein), *Ca_14797* (ZOS9-17 - C2H2 zinc finger protein), *Ca_07131* (homeobox and START domain containing protein), *Ca_10504* (MYB family transcription factor) and *Ca_27036* (PHD-finger domain containing protein) harbored either promoter or non-synonymous DNA polymorphisms.

**Figure 6:**
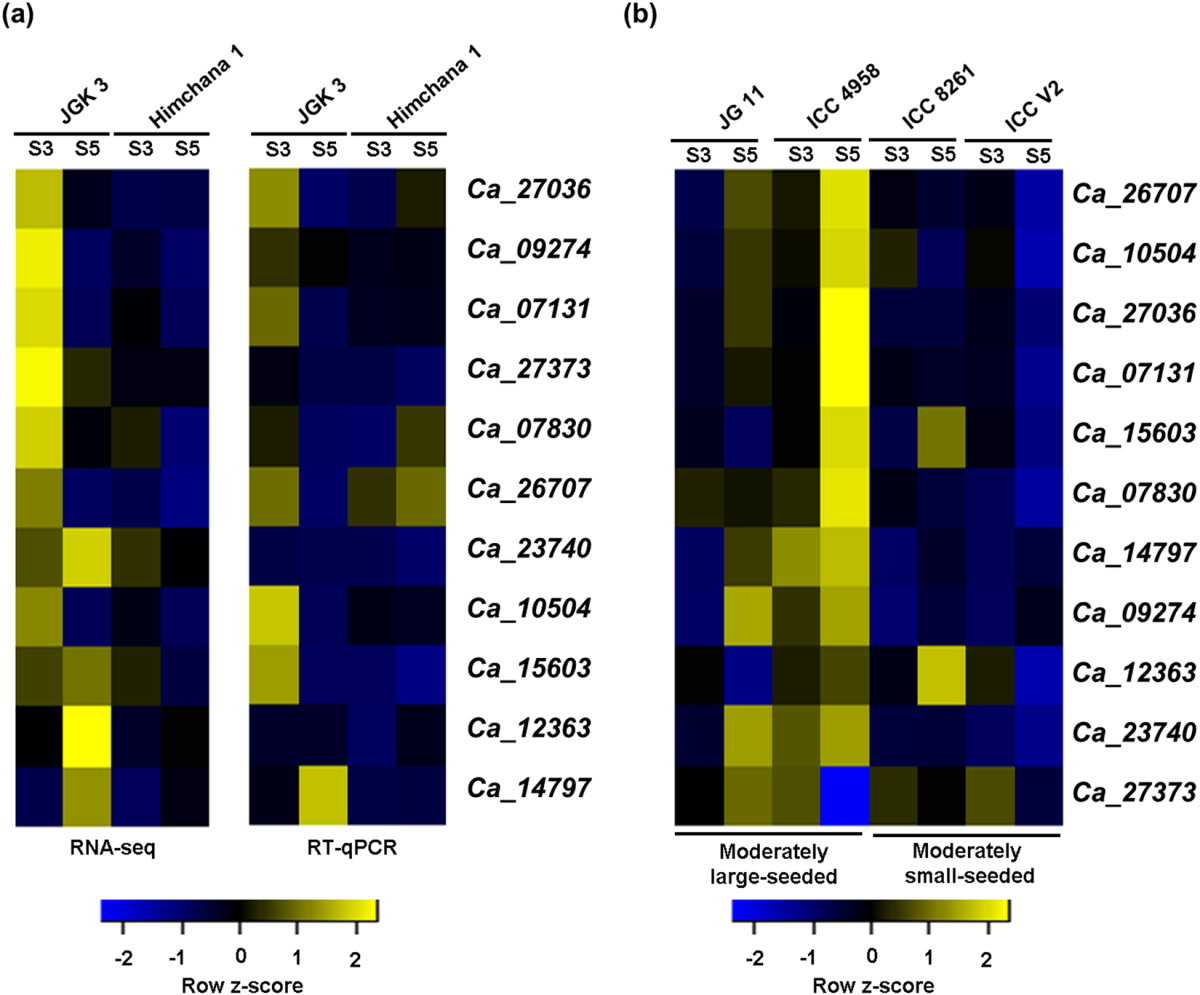
Validation and differential expression analysis of genes involved in seed size/weight determination via RT-qPCR. (**a**) Expression profile of the genes at late-embryogenesis (S3) and mid-maturation (S5) stages of seed development in JGK 3 and Himchana 1 cultivars revealed from RNA-seq and RT-qPCR is shown in the left and right panels, respectively. (**b**) Differential expression of these genes at S3 and S5 stages of seed development in two moderately large desi-type (JG 11 and ICC 4958) and moderately small-seeded kabuli-type (ICC V2 and ICC 8261) chickpea genotypes via RT-qPCR analysis is shown. Color scales at the bottom represent row-wise z-score.

Among the 22 genes, we randomly selected 11 genes to validate and analyze their differential expression between the small (Himchana 1) and large-seeded (JGK 3) chickpea cultivars via RT-qPCR at late-embryogenesis (S3) and/or mid-maturation (S5) seed stages. RT-qPCR analysis showed higher expression of these genes at S3 and/or S5 stages of seed development in JGK 3 as compared to Himchana 1, as revealed by RNA-seq (Fig. 6a). To further validate the higher expression of these genes in other large-seeded genotypes, we performed RT-qPCR analysis at the corresponding seed development stages in two additional moderately large-seeded desi-type (ICC 4958 and JG 11 with 100 seeds weight of 28.46 ± 0.94 g and 25.3 ± 0.17 g, respectively) and two moderately small-seeded kabuli-type (ICC V2 and ICC 8261 with 100 seeds weight of 26.4 ± 0.6 g and 33.51 ± 1.95 g, respectively) chickpea genotypes. Overall, higher expression of most of these genes was found in the moderately large-seeded desi-type chickpea accessions as compared to the moderately small-seeded kabuli-type chickpea accessions (Fig. 6b). However, among the two small-seeded kabuli-type chickpea accessions, expression of a few of these genes was higher in ICC 8261 especially at S5 stage of seed development as compared to ICC V2 (Fig. 6b), which may be due to higher seed weight in ICC 8261. Although, the seed weight of the large-seeded desi-type and small-seeded kabuli-type chickpea accessions was comparable, higher expression of these genes in the large-seeded desi-type (ICC 4958 and JG 11) and large-seeded kabuli-type (JGK 3) chickpea genotypes suggested that these genes may play important role in seed size/weight determination in chickpea type (desi or kabuli) specific manner. Previous study showed no distinct lineages between desi-type and kabuli-type chickpeas based on SNP data analysis from 93 accessions including 19 desi-type and 10 kabuli-type^65^, suggesting that both desi-type and kabuli-type chickpeas might have coevolved. But how seed size/weight is determined in different chickpea types (desi and kabuli) remains to be investigated further.

In conclusion, we identified DNA polymorphisms that differentiated small and large-seeded chickpea cultivars via genome resequencing. Integrated analyses of DNA polymorphisms and gene expression revealed seed-specific genes harboring DNA polymorphisms in promoter, non-synonymous and large-effect DNA polymorphisms, which may determine seed size/weight. Further, the influence of DNA polymorphisms on seed-specific genes involved in cell growth and division, genes associated with QTLs and those encoding transcription factors, was revealed. We detected 22 candidate genes showing higher expression at late-embryogenesis and/or mid-maturation stage of seed development in the large-seeded cultivar(s), which might be due to the DNA polymorphisms among them. Functional validation of these genes will be important to understand their precise role in seed size/weight determination. Altogether, this study provides important resource of DNA polymorphisms for large-scale genotyping for various applications and highlighted a few candidate genes that may play role in seed size/weight determination in chickpea.

## Methods

### Genomic DNA extraction and sequencing

Genomic DNA was isolated from leaves of four chickpea (*Cicer arietinum* L.) cultivars, i.e., Himchana 1, Pusa 362, JGK 3 and PG 0515 using Qiagen DNeasy kit (Qiagen, GmbH, Hilden). The extracted DNA was checked in agarose gel electrophoresis and quantified using Qubit 2.0 Fluorimeter (Invitrogen Life Technologies, Eugene, Oregon). The sequencing libraries were prepared as per manufacturer’s recommendations for paired-end reads to generate 90 base long reads using Illumina Hiseq 2000 platform (Illumina Technologies). The raw files were processed to remove low quality reads and adaptor/primer contamination using NGS QC toolkit (v2.3)^66^.

### Read mapping

The high-quality filtered reads were mapped on the kabuli chickpea reference genome (v1.0) using BWA software (v6.2). The redundant reads were removed to retain only the uniquely mapped reads. Furthermore, we retained only high-quality aligned reads with mapping score ≥30. The coverage of the genome was estimated using SAMtools (v0.1.16).

### Identification of DNA polymorphisms

The filtered alignment files were used for the identification of four types of variants (SNPs, InDels, MNPs and complex) using FreeBayes software (v0.8.7). It has been shown that FreeBayes detects InDels with high precision^67,68^. We identified DNA polymorphisms by mapping the aligned files to the kabuli reference genome using min-mapping-quality 30 (default), min-coverage 10, min-alternate-fraction 0.9 parameters. In addition, we removed the DNA polymorphisms, if three or more of them were present within any 10-bp window as described^69^. To analyze the genomic distribution of DNA polymorphisms, we calculated their frequency per 100 kb window for each chromosome. To annotate the DNA polymorphisms, we mapped their position on the kabuli genome annotation using customized perl script. SNP effect predictor (SnpEff, v3.1 h) was used to identify the effect of all types of DNA polymorphisms. To identify the genes harboring significantly high frequency of DNA polymorphisms, the frequency of DNA polymorphisms (SNPs, InDels, MNPs and complex variants) per kb in the genic regions were plotted using boxplot. Genes harboring DNA polymorphisms above the third quartile in the boxplot were considered as highly polymorphic genes.

### Gene ontology analysis

Functional categorization of genes harboring DNA polymorphisms was performed using eukaryotic orthologous groups (KOG) based on the sequence homology. Further, enrichment analysis of gene ontology terms was performed via BiNGO plug-in of Cytoscape (version 3.3.0) with *p*-value ≤ 0.05. The best Arabidopsis match to the chickpea genes based on sequence similarity was used for finding enrichment of gene ontology terms as described earlier^6^.

### RNA-seq based gene expression analysis

Gene expression analysis was performed using the transcriptome data for seven stages (S1–S7) representing embryonic (S1–S3), mid-maturation (S4 and S5) and late maturation (S6 and S7) stages of seed development in Himchana 1 and JGK 3 from our previous study^6^. Differential gene expression between the two cultivars at a particular stage of seed development was determined using Cuffdiff, as described earlier^6^. Fold change of at least two and corrected *q*-value of ≤0.05 were set as criteria to identify differentially expressed genes. Differential gene expression was plotted via heatmaps using row-wise z-score based on FPKM expression values. To identify seed-specific genes, we calculated stage specificity index^70^ based on the transcriptome data from14 seed stages and 15 vegetative and/or floral tissues as described in previous studies^6,50^. Genes showing higher expression in any stage of seed development and >0.6 tissue specificity index were identified as seed-specific genes.

### Tissue collection and RT-qPCR analysis

Seeds corresponding to late-embryogenesis (S3) and mid-maturation (S5) stages of seed development from Himchana 1, JGK 3, ICC 8261, ICC V2, ICC 4958 and JG 11 were collected from field-grown plants as described in previous study^6^. Seeds were snap frozen in liquid nitrogen and stored at −80 °C until use. Total RNA was extracted using TRI reagent (Sigma Life Science, St. Louis, MO) as per manufacturer’s instructions. RNA isolated from the S5 stage was further purified with additional phenol-chloroform step followed by ethanol precipitation to remove starch content and other secondary metabolites. Gene-specific primers were designed using Primer Express (v3.0). Following cDNA synthesis, qPCR was performed for at least two biological replicates and each biological replicate was analyzed in three technical replicates as described earlier^6^. *Elongation factor 1 alpha* was used as internal control for normalization and expression of the genes at S3 and/or S5 stages of seed development was analyzed as described earlier^6^. List of primers used for validation of differential gene expression has been provided in Supplementary Table S5.

### SNP validation

A total of 48 SNPs located in 20 genes were randomly selected for validation via Sequenome MassARRAY iPLEX platform (Agena Bioscience, San Diego, USA) as described earlier^71^. To validate these SNPs, forward and reverse primers spanning the polymorphic sites and the extension primer that probe the SNP position were designed using Sequenom’s MassARRAY Designer software. The list of primers used to validate SNPs has been provided in Supplementary Table S6. Briefly, the genomic DNA from the four chickpea cultivars (Himchana 1, Pusa 362, JGK 3 and PG 0515) was diluted to 5 ng/µl concentration using Tris-EDTA buffer. About 10 ng of genomic DNA was used for PCR amplification. The PCR products were processed to remove the unincorporated dNTPs using shrimp alkaline phosphatase. Purified PCR products were used as template in iPLEX reaction that discriminates different alleles. A site-specific primer (extension primer) which binds to one base upstream of the polymorphic site and mass-modified ddNTPs were added to the cocktail of iPLEX reaction. Incorporation of mass-modified ddNTP at the polymorphic site followed by its detection with mass spectrometry enabled to identify the base at the polymorphic site in the four chickpea cultivars. Finally, the base calls identified via mass spectrometry and the genome resequencing were compared.

### Identification of QTL associated genes

Co-ordinates of QTLs and/or genes associated with seed size/weight in chickpea were retrieved from the previous studies^6,20–25^. We identified the genes located within these QTLs using BedTools.

## Electronic supplementary material

Supplementary Data

Supplementary File 1

Supplementary File 2
