# Supplementary material for: Genome-wide discovery of DNA polymorphisms among chickpea cultivars with contrasting seed size/weight and their functional relevance
Source: Sci Rep. 2018 Nov 14;8:16795. doi: 10.1038/s41598-018-35140-w (PMC6235875; doi:10.1038/s41598-018-35140-w)
Supplement: Supplementary file 1 — Supplementary Data [file 41598_2018_35140_MOESM1_ESM.pdf]

Subject Area: Plant Biology

*Correspondence and requests for material should be addressed to

M.J. (mjain@jnu.ac.in)

Genome-wide discovery of DNA polymorphisms among chickpea cultivars with contrasting seed size/weight and their functional relevance

Mohan Singh Rajkumar[1], Rohini Garg[2] and Mukesh Jain[1, 3]*

[1] School of Computational & Integrative Sciences, Jawaharlal Nehru University, New Delhi - 110067, India,

[2]Department of Life Sciences, School of Natural Sciences, Shiv Nadar University, Gautam Buddha Nagar, Uttar Pradesh - 201314, India and

[3]National Institute of Plant Genome Research (NIPGR), Aruna Asaf Ali Marg, New Delhi 110067, India.

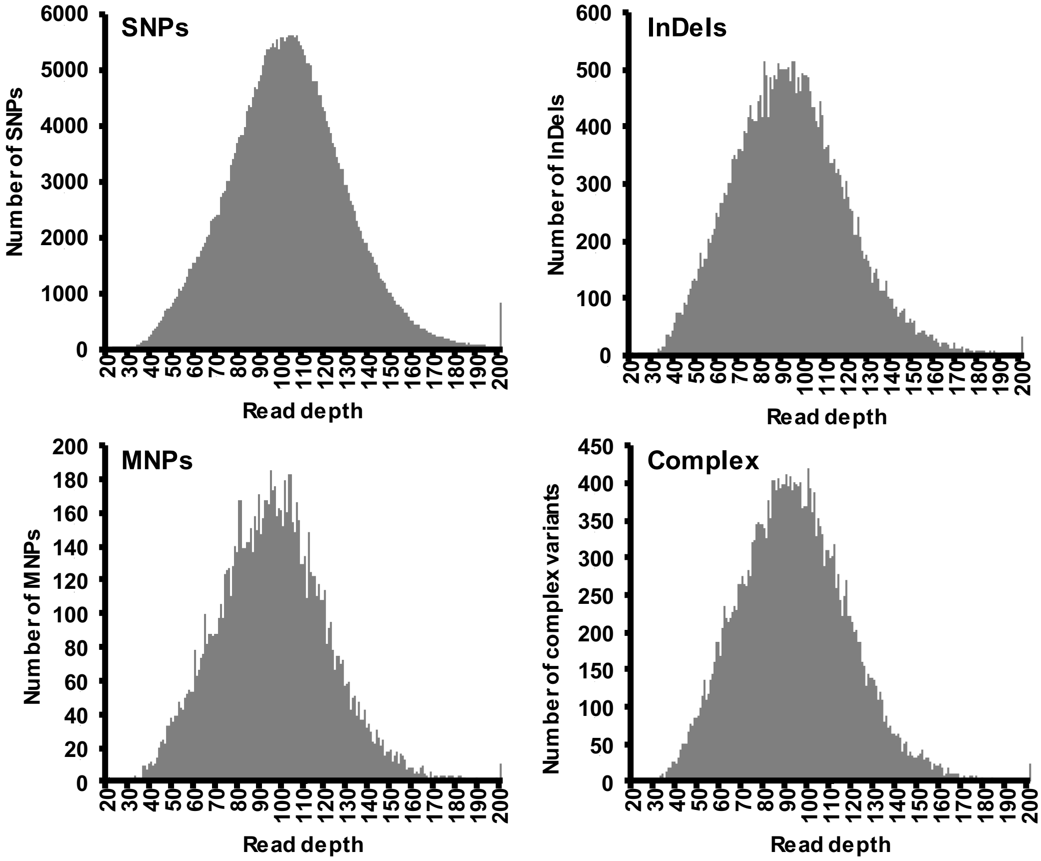

Supplementary Figure S1. Number of different types of DNA polymorphisms (SNPs, MNPs, InDels and complex variants) at different read depths are shown in bar graphs.

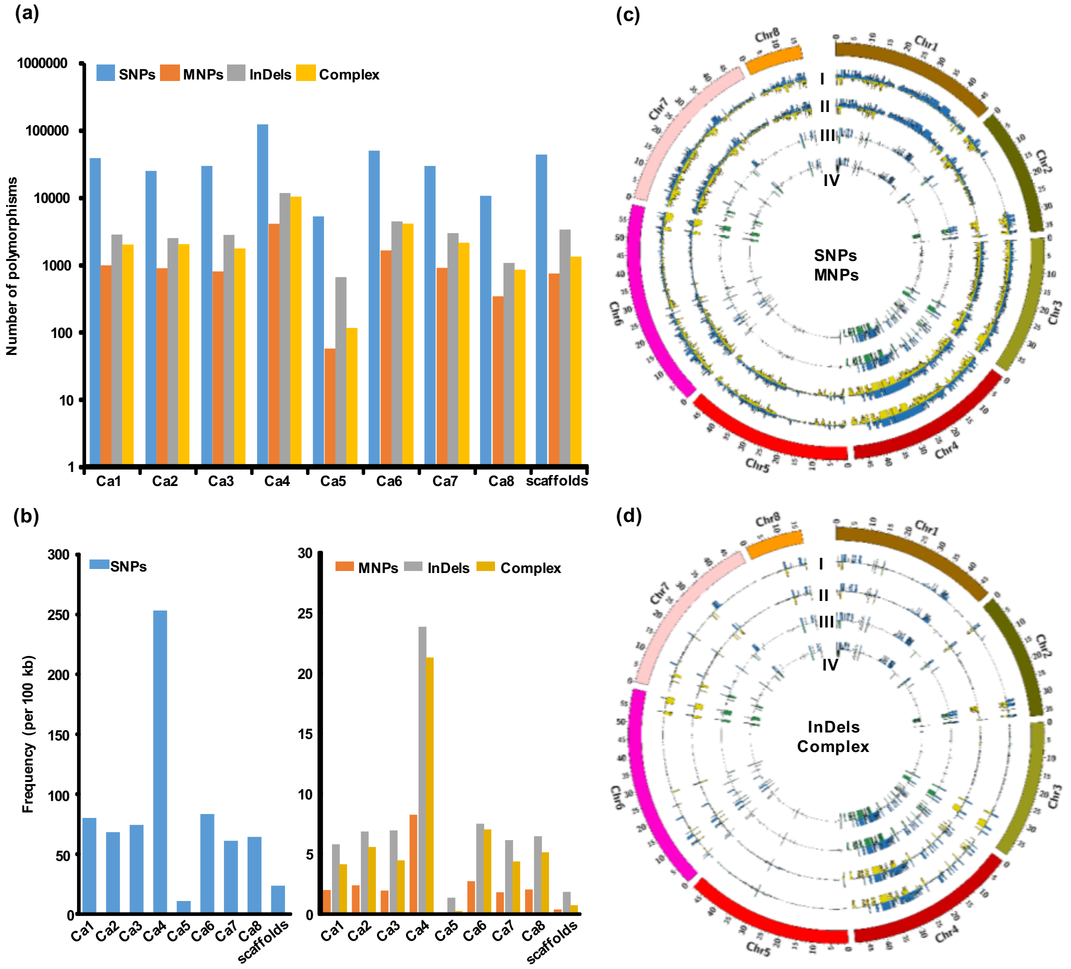

Supplementary Figure S2. The number and frequency of DNA polymorphisms identified between small and large-seeded chickpea cultivars. (a, b) Number (a) and frequency (b) of DNA polymorphisms (SNPs, InDels, MNPs and complex variants) identified on different chickpea chromosomes and scaffolds are shown in bar graphs. (c, d) Frequency of SNPs, MNPs, InDels and complex variants on the chickpea genome (per 100 kb) is shown using Circos diagrams. The outermost circle represents eight individual chromosomes of chickpea with different colors. (c) Circles I and II represent SNPs and circles III and IV represent MNPs. The frequency of SNPs in the outward and inward bars in circle I represent the SNPs identified in Himchana 1/JGK 3 and Pusa 362/JGK 3, respectively. Similarly, outward and inward bars of circle II represent SNPs identified in Himchana 1/PG 0515 and Pusa 362/PG 0515, respectively. Likewise, outward and inward bars in the circles III and IV represent MNPs (d) Circles I and II represent InDels and circles III and IV represent complex variants and the bars in the outward and inward directions are as described for (c).

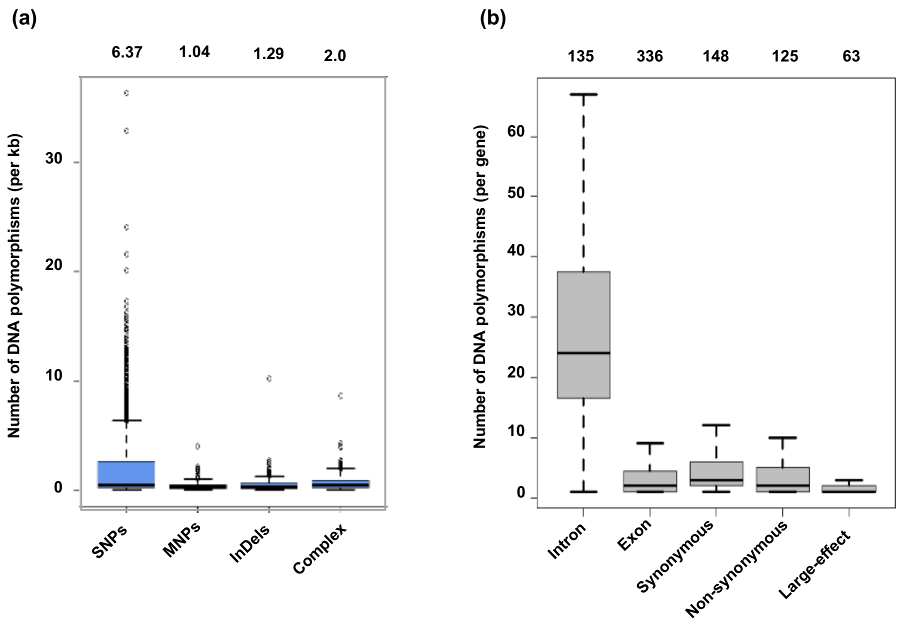

Supplementary Figure S3. Genes harboring significantly high frequency of DNA polymorphisms. (a) Frequency of DNA polymorphisms per kb of genic regions is shown via boxplot. Dots lying above the third quartile show significantly high frequency of DNA polymorphisms (outliers). Numbers above the boxplot indicate third quartile values for different types of DNA polymorphisms. (b) Number of significantly high frequency of DNA polymorphisms (per gene) in the exonic and intronic regions are shown via boxplot. Likewise, number of significantly high frequency of DNA polymorphisms associated with synonymous, non-synonymous and large-effect changes are shown. Numbers above the boxplot indicate total number of genes harboring the DNA polymorphisms.

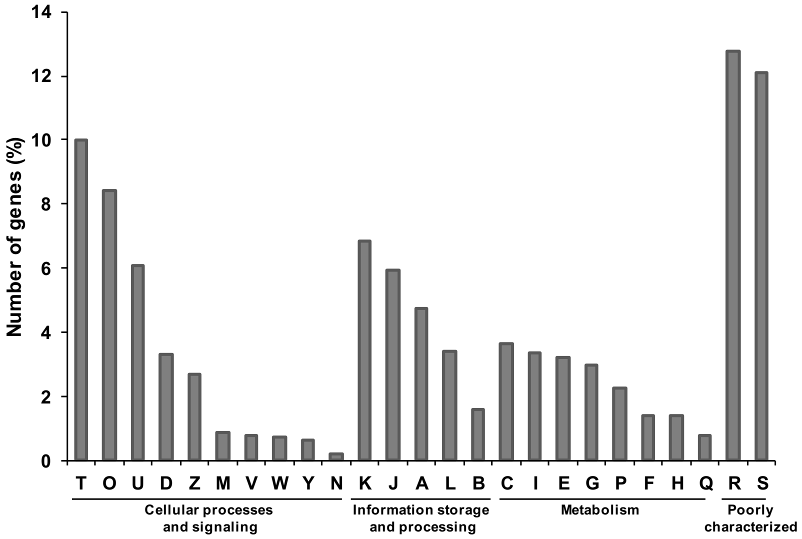

Supplementary Figure S4. Functional categorization of genes harboring significantly high frequency of DNA polymorphisms in the genic region (outliers) via eukaryotic KOG analysis. The percentage of genes in specific KOG classes are shown using different alphabets for different categories. Cellular processes and signaling (T, signal transduction mechanisms; O, post-translational modification, protein turnover, chaperons; U, intracellular trafficking, secretion, and vesicular transport; D, cell cycle control, cell division, chromosome partitioning; Z, cytoskeleton; M, cell wall/membrane/envelope biogenesis; V, defense mechanisms; W, extracellular structures; Y, nuclear structure; N, cell motility), Information storage and processing (K, transcription; ; J, translation, ribosomal structure and biogenesis; A, RNA processing and modification; L, replication, recombination and repair; B chromatin structure and dynamics), Metabolism (C, energy production and conversion; I, lipid transport and metabolism; E, amino acid transport and metabolism; G, carbohydrate transport and metabolism; P, inorganic ion transport and metabolism; F, nucleotide transport and metabolism; H, coenzyme transport and metabolism; Q, secondary metabolites biosynthesis, transport and catabolism) and poorly characterize functions (R, general function prediction only and S, function unknown).

Supplementary Table S1. Mapping efficiency of reads in small (Himchana 1 and Pusa 362) and large-seeded (JGK 3 and PG 0515) chickpea cultivars.

	**Pusa 362**	**Himchana 1**	**JGK 3**	**PG 0515**
Total reads	202121350	204631846	189723748	213075762
Total mapped reads	199251701	202668256	187826765	210361412
Percentage of mapped reads	98.58	99.04	99.0	98.73
Total uniquely mapped reads	117520515	125826115	116105754	131679311
Percentage of uniquely mapped reads	58.14	61.49	61.2	61.8

Supplementary Table S2. Percentage coverage of uniquely mapped reads for small (Himchana 1 and Pusa 362) and large-seeded (JGK 3 and PG 0515) chickpea cultivars.

Chromosome	Pusa 362	Himchana 1	JGK 3	PG 0515
Ca1	88.93	88.68	88.77	88.85
Ca2	87.85	87.98	87.66	87.97
Ca3	90.94	91.09	90.91	91
Ca4	90.94	91.95	90.67	90.71
Ca5	87.23	87.2	87.11	87.17
Ca6	88.93	89.23	89.1	89.12
Ca7	90.86	91.01	90.84	90.95
Ca8	86.52	86.52	86.58	86.6
Genome coverage (%)	83.71	83.9	83.6	83.68

Supplementary Table S3. Number of DNA polymorphisms (SNP, MNPs, InDels and complex variants) associated with large-effect.

	**SNPs**	**MNPs**	**InDels**	**Complex**
Splice site acceptor	24	0	1	2
Splice site donor	21	0	9	0
Start lost	16	0	0	6
Stop gained	82	6	0	10
Stop lost	22	0	1	9
Codon deletion	0	0	87	12
Codon insertion	0	0	15	0
Frame shift	0	0	170	13
Total	165	6	283	52

Supplementary Table S4. Validation of SNPs identified using genome sequencing with mass spectrometry.

		Resequencing				Mass spectrometry					
**Chromosome**	**Position**	**1**	**2**	**3**	**4**	**1**	**2**	**3**	**4**	**Gene ID**	**Region/effect**
Ca1	424771	G	G	T	T	G	G	T	T	Ca_00047	non-synonymous
Ca1	425388	T	T	A	A	T	T	A	A	Ca_00047	upstream
Ca1	425453	G	G	A	A	G	G	A	A	Ca_00047	upstream
Ca1	425553	C	C	A	A	C	C	C	A	Ca_00047	upstream
Ca1	425598	A	A	G	G	NA	A	G	G	Ca_00047	upstream
Ca1	1849192	C	C	G	G	C	C	G	G	Ca_00232	non-synonymous
Ca1	1850262	T	T	G	G	T	T	G	G	Ca_00232	upstream
Ca1	1850278	C	C	G	G	C	NA	G	G	Ca_00232	upstream
Ca1	1850343	G	G	A	A	G	G	A	A	Ca_00232	upstream
Ca1	16187950	C	C	T	T	C	C	T	T	Ca_06937	upstream
Ca1	16188854	T	T	G	G	T	T	G	G	Ca_06937	upstream
Ca1	16184780	C	C	T	T	C	C	T	T	Ca_06937	synonymous
Ca1	16184848	C	C	G	G	C	C	G	G	Ca_06937	non-synonymous
Ca1	16184909	T	T	C	C	T	T	C	C	Ca_06937	synonymous
Ca1	16184990	C	C	T	T	NA	C	T	T	Ca_06937	synonymous
Ca1	16187557	G	G	C	C	G	G	C	C	Ca_06937	upstream
Ca1	16187844	T	T	A	A	T	T	A	A	Ca_06937	upstream
Ca3	18005043	G	G	T	T	G	G	T	T	Ca_21133	non-synonymous
Ca3	31413666	A	A	G	G	A	A	G	G	Ca_12237	upstream
Ca4	4958708	A	A	G	G	A	G	G	G	Ca_03762	non-synonymous
Ca4	4962647	T	T	C	C	T	T	C	C	Ca_03762	upstream
Ca4	11869382	G	G	A	A	G	G	A	A	Ca_04409	upstream
Ca4	12566986	T	T	C	C	C	C	C	C	Ca_04479	upstream
Ca4	12568458	C	C	T	T	C	T	T	T	Ca_04479	synonymous
Ca4	12568476	T	T	C	C	T	C	C	C	Ca_04479	synonymous
Ca4	12569220	T	T	C	C	T	T	C	C	Ca_04479	synonymous
Ca4	16665206	G	G	C	C	G	G	C	C	Ca_05457	synonymous
Ca4	16665879	C	C	T	T	C	T	T	T	Ca_05457	non-synonymous
Ca4	16671857	G	G	A	A	G	A	A	A	Ca_05457	synonymous
Ca4	17137187	G	G	A	A	G	G	A	A	Ca_05411	upstream
Ca4	17348041	G	G	A	A	G	G	A	A	Ca_05389	upstream
Ca4	24557836	C	C	T	T	C	C	T	T	Ca_20862	upstream
Ca4	24559011	T	T	G	G	T	T	G	G	Ca_20862	upstream
Ca5	27361668	T	T	G	G	T	T	G	G	Ca_08907	non-synonymous
Ca5	27363412	A	A	G	G	A	A	G	G	Ca_08907	upstream
Ca5	32597880	T	T	C	C	NA	T	C	C	Ca_04966	synonymous
Ca5	32600023	A	A	G	G	A	A	G	G	Ca_04966	upstream
Ca6	13131131	G	G	T	T	G	G	T	T	Ca_05086	synonymous
Ca8	8938796	G	G	A	A	G	G	A	A	Ca_11524	upstream
Ca8	8938891	T	T	C	C	T	T	C	C	Ca_11524	upstream
Ca8	8939190	G	G	A	A	G	G	A	A	Ca_11524	upstream
Ca8	8939219	C	C	T	T	C	C	T	T	Ca_11524	upstream
scaffold1128	419574	T	T	A	A	A	T	A	A	Ca_23740	upstream
scaffold1128	419933	T	T	A	A	T	T	A	A	Ca_23740	upstream
scaffold4777	6305	T	T	A	A	T	T	A	A	Ca_26708	non-synonymous
scaffold4777	6791	C	C	T	T	C	C	T	T	Ca_26707	upstream
scaffold4777	5884	T	T	C	C	T	T	C	C	Ca_26709	upstream
scaffold4777	14064	A	A	T	T	A	A	T	T	Ca_26707	large-effect

Labels 1, 2, 3 and 4 represent Himchana 1, Pusa 362, JGK 3 and PG 0515, respectively. NA represents no base call in mass spectrometry.

Supplementary Table S5. List of primers used for RT-qPCR validation.

Gene identifier	Forward primer (5'-3')	Reverse primer (5'-3')
Ca_12363	ACAAGAAGTAGCACAAAGCAATGAA	GTGCAAAAGGTGGTCTTATCATGAG
Ca_14797	TCTCCAGGCGTGATAGTTTCATC	GAAGGGTGTCTTGAATTTTCATGTG
Ca_07131	TGCAAAGTACGTAGGTAAGCCAGTA	GAGGACGAGGAGGTACAGATGAAG
Ca_10504	AAGTGCTCGTCTTGAAGCTGAA	GTGATTATGAATTGAATGTGAACGAA
Ca_15603	GATGGCACAAAGTGGCTGAAA	TCCTTGTACTGATTCAACACATCAAC
Ca_27036	CCAGAGCACCGATGCTAACC	AATTGCAACCGTCGCAGAA
Ca_27373	GCTGTATGAACCATTCCTGTTGTC	TGGCCATCTCTATCCTCATCTCTT
Ca_07830	ACCGTCACTGGAAATTTTGGA	CAGCCACAGAAACAGCAGCTT
Ca_23740	GGACGGTTGAGTTGCATCAA	GTCCAGCCTTATCAACTCCAAGTT
Ca_26707	TGTATGGCTTATTGGTTGTCCAA	GGTGGTTTTCGTGCTGGTACTC
Ca_09274	CGTCTATGGCCAATGGTAAAGC	ACCATTTTAGAACATTGACGACCTT

Supplementary Table S6. List of primers used for SNP validation.

Chromosome	Position	Forward primer (5'-3')	Reverse primer (5'-3')	Extended primer (5'-3')
Ca1	424771	ACGTTGGATGCTTTGCCAGCACAAGAAGAG	ACGTTGGATGCATTCTTGACCAAGCTGAGG	CGCATGAGTAAGGAAGTTCAA
Ca1	425388	ACGTTGGATGGGTAAGTGGTAACCAATCTC	ACGTTGGATGGAATTAAGGTACTCTTGATTG	TAAGGTACTCTTGATTGATTTATTATT
Ca1	425453	ACGTTGGATGTGGTTGAAACCATCTCTGGC	ACGTTGGATGCGATTTTCGAGAGAAAGAGG	TGCCATCTCTGGCCAACATT
Ca1	425553	ACGTTGGATGCTCGAAAATCGAATGCAAGAG	ACGTTGGATGGTTGTATGACTGAGTGAAAGC	GGGAAGTGAAAGCACTTTGATGA
Ca1	425598	ACGTTGGATGGTATCCTCTCATCAAAGTGC	ACGTTGGATGTAAACCATATTGAAGAGCC	CAGTCATACAACCTATATTTTATCT
Ca1	1849192	ACGTTGGATGACCAAACCCATTTTCATGCC	ACGTTGGATGTTTGAGCCAAATGTGGAAGC	CCCCGTCACTGACCTTAGTTACAAT
Ca1	1850262	ACGTTGGATGCACAACAAGGTGTTCTTCCC	ACGTTGGATGTCTCTTCCATACCTTCAACC	TGTTCTTCCCATAGCAAAT
Ca1	1850278	ACGTTGGATGCACAACAAGGTGTTCTTCCC	ACGTTGGATGTCTCTTCCATACCTTCAACC	CATACCTTCAACCAAACTAATT
Ca1	1850343	ACGTTGGATGTGGTTGAAGGTATGGAAGAG	ACGTTGGATGTTCCATTACGGGTACCCTAC	AGGAAATTACGGGTACCCTACAAACAAT
Ca1	16183993	ACGTTGGATGAGTGTGGTTTTTTCCTTTC	ACGTTGGATGATCCTTTCACATACACACAC	ACATTTTATTTAAATTTTCTTCTTTACT
Ca1	16184114	ACGTTGGATGCGTTCATTTTTATTTGAGATG	ACGTTGGATGCTAATTTCTAATTAGTTAGAG	ATTTTTATTTGAGATGTAAAATTAGTTA
Ca1	16184780	ACGTTGGATGTGACACACCTAGGAGTCTTG	ACGTTGGATGTGTGGAACAGGGGACATTTG	GGATTATGCTATTGGTTTAGG
Ca1	16184848	ACGTTGGATGTCAAATGTCCCCTGTTCCAC	ACGTTGGATGTATCGACAAGGTCGTGTCTC	CCACAATCTTAATCTCAAGTG
Ca1	16184909	ACGTTGGATGGATTTATCTGAGACACGACC	ACGTTGGATGTGTGTGTACGAGGATTGTTG	ACAGGTACGAGGATTGTTGTTTAAC
Ca1	16184990	ACGTTGGATGCCCTTTGAGATTTAGTTCCC	ACGTTGGATGATGCAGATGTGGCTGAATAC	GGTTCAGATACAACAACAATTCCAGG
Ca1	16187557	ACGTTGGATGACGGTGTCTCTTGGAATAGG	ACGTTGGATGCCTTAGCTCTTTTTTTCTCC	TCATCTACAAACAAACACTTCTT
Ca1	16187844	ACGTTGGATGCTACAACTACTAGTTTGTACC	ACGTTGGATGATTTCTATGATTTAATCGAC	CTATGATTTAATCGACAAAATCTTAA
Ca3	18005043	ACGTTGGATGCTTTCCACTGAAGGTTTGTC	ACGTTGGATGAGTGACCGACGTACATCAAC	CTGAAGGTTTGTCCAAACT
Ca3	31413666	ACGTTGGATGGGGCTGCAGTTCAGGAAAAT	ACGTTGGATGTACCCTTGTTGTGTTTTGAC	TTTGGACAACTATGACAAATACGGT
Ca4	4958708	ACGTTGGATGGGTTTGATCTTTCCTTTGAG	ACGTTGGATGAGAACAAGTTAGTCTTGCGG	TGGTCTTGCGGATTGGG
Ca4	4962647	ACGTTGGATGGGAGCAGAAGTAGATTCTCG	ACGTTGGATGTGCATTGTCGTTATCGCCTC	TGTGGGATCCTTGCATGGAAAGTCTT
Ca4	11869382	ACGTTGGATGGCATGTCTATTGTATGAGCG	ACGTTGGATGAAATTCACCAAAAGTAATAC	CATTTGTAGTGTCCACG
Ca4	12566986	ACGTTGGATGAAATATATTTTCAGGCCAC	ACGTTGGATGTGAAAACCCAGGAGGTCAGC	CCGGAGGTCAGCTTGAAA
Ca4	12568458	ACGTTGGATGATCCTAATGTTCACCCTGGC	ACGTTGGATGCAGAGAGGGTTTTTGCTGAG	ACCCGCTGAGTTTGCTGATGTT
Ca4	12568476	ACGTTGGATGTGGCGAGATATCCATCTCTG	ACGTTGGATGATACCGGCGCAATTCCAGAG	GCCCCTAACATCAGCAAACTCAG
Ca4	12569220	ACGTTGGATGTGAAGGCTTCTGCTAATGTC	ACGTTGGATGCCTACTCACAAGCTACAACG	TACGAATAAGTCTAGCACCGACGCGATC

Ca4	16665206	ACGTTGGATGTCTCTTCTCCCTTTACCGTC	ACGTTGGATGCTATCGAAGATCTGCACGAC	CCGACGTGCTTCTCCAT
Ca4	16665879	ACGTTGGATGGTTTGCAGATTCAAACAAAG	ACGTTGGATGGACTGCAATGATGAACTGGG	TTTTGCAGATTCAAACAAAGATGATA
Ca4	16671857	ACGTTGGATGGTTGCCTATAAACTAAGCACC	ACGTTGGATGGAGTCCTGACTTGGTACTAC	CTGAAGCTTCCGAGG
Ca4	17137187	ACGTTGGATGAAAATTTGGAACACATGTTT	ACGTTGGATGAGCCCTAAATATTGTGTACC	GGTGAAAAATTTTGAACTTCGTATCG
Ca4	17348041	ACGTTGGATGGGCCATGTGTCATTGTTTTG	ACGTTGGATGACACGTGATAATGGTTGTTG	GTTTGCACCGGTTGA
Ca4	24557836	ACGTTGGATGGCCTTCACTAAAGGAGAACC	ACGTTGGATGGTGATCGCATACCACTTTTC	GGGACTTCACTAAAGGAGAACCTCAAGG
Ca4	24559011	ACGTTGGATGGATGATTATGACTTGGAAG	ACGTTGGATGGAGGAATATAAAAACAAGTC	CCTGACTTGGAAGATGAAGA
Ca5	27361668	ACGTTGGATGTGATCGTAATACAGCGCGTG	ACGTTGGATGTATCTTACGAAGCGCGCGTG	GCACCGGTAACCGTTT
Ca5	27363412	ACGTTGGATGTGCTATGGTTTTGGTATGGG	ACGTTGGATGCACCCATGAATCCATTACTC	ATACACTAGGCAAAGAGAATGA
Ca5	32597880	ACGTTGGATGAACTCCGCCGTATTAAACGC	ACGTTGGATGATTGCAGGTAACGGAGGAAG	CCCCCGCCGCACACGCACT
Ca5	32600023	ACGTTGGATGGTCGTCTCTATCGTTTGTTTC	ACGTTGGATGCTTTCAGAAACGCAAGGGCA	CGTTTGTTTCAGAAGCG
Ca6	13131131	ACGTTGGATGCATGATGTTCTCTTCGGTCC	ACGTTGGATGAGGACATTGGAGTGAAGGAG	CCTTGAAGGAAGATTTGTAACATC
Ca8	8938796	ACGTTGGATGAGATGTATTTAATATTCGAC	ACGTTGGATGCGAAAATAACACAATATCAC	GATGTATTTAATATTCGACACCAAC
Ca8	8938891	ACGTTGGATGGTGATATTGTGTTATTTTCG	ACGTTGGATGTGGTAACAGGGTAAACAATC	CCACCAGGGTAAACAATCTTTTTACTAC
Ca8	8939190	ACGTTGGATGGCAACGTGAATGTCTTGCTG	ACGTTGGATGTGGACATCACAACATCGCTC	TGCTGAGAAGTTTACGGG
Ca8	8939219	ACGTTGGATGCTTGCTGAGAAGTTTACGGG	ACGTTGGATGGCGGGTTTGGTTATCACTAC	AGGTGATGAAAATGAGCGA
scaffold1128	419574	ACGTTGGATGATCACTTCAACTCCTCAAAC	ACGTTGGATGAGTTACATATATGTGATTTC	TCAACTCCTCAAACTACCTTA
scaffold1128	420593	ACGTTGGATGGATTTCACTCATCTTTGCTC	ACGTTGGATGAAGAAAGGAGTGGCATAGGG	AGCTTTGCTCAATTCACCTT
scaffold4777	6305	ACGTTGGATGGAATTTTAATTGTCCCCAAC	ACGTTGGATGGTCTTGTTGTTGATGATCC	CCCCCAATTGTCCCCAACAAATTATA
scaffold4777	6791	ACGTTGGATGGACTCGACATATCTTCCAAC	ACGTTGGATGGCTGAAAACTTCTCAATCTC	CCTCGACATATCTTCCAACATTTAATAA
scaffold4777	6908	ACGTTGGATGGAGAATTACTTCATTGCAC	ACGTTGGATGGCTGCTCAAACCTTATATTCC	AACATTATTTTAAGTTTTGGAGATTGAA
scaffold4777	14347	ACGTTGGATGGTTCTCTAGTATGACACGAG	ACGTTGGATGTAATTGCCTCCATAACTCCC	CCTCCATAACTCCCTGTAAA
